# Cerebrovascular Accident Secondary to Paradoxical Embolism Following Arteriovenous Graft Thrombectomy

**DOI:** 10.1155/2012/183730

**Published:** 2012-09-25

**Authors:** Jolina Pamela Santos, Zaher Hamadeh, Naheed Ansari

**Affiliations:** Renal Division, Department of Medicine, Jacobi Medical Center, Albert Einstein College of Medicine, 1400 Pelham Parkway South, Bronx, New York, NY 10461, USA

## Abstract

Thrombectomy is a common procedure performed to declot thrombosed dialysis arteriovenous fistula (AVF) or arteriovenous graft (AVG). Complications associated with access thrombectomy like pulmonary embolism have been reported, but paradoxical embolism is extremely rare. We report a case of a 74-year-old black man with past medical history significant for end-stage renal disease (ESRD), atrial fibrillation on anticoagulation with warfarin, who presented to our hospital with lethargy, aphasia, and right-sided hemiparesis following thrombectomy of a clotted AVG. Computed tomography (CT) scan of brain showed a hypodensity within the left posterior parietal lobe. INR was 2.0 on admission. Echocardiogram revealed a normal sized left atrium with no intracardiac thrombus, and bubble study showed the presence of right-to-left shunting. These findings suggest that the stroke occurred as a result of an embolus originating from the AVG. Paradoxical cerebral embolism is uncommon but can occur after thrombectomy of clotted vascular access in ESRD patients. Clinicians and patients should be aware of this serious and potentially fatal complication of vascular access procedure.

## 1. Introduction

Hemodialysis vascular accesses are commonly complicated by thrombosis. Thrombolysis using percutaneous endovascular techniques by means of mechanical devices such as balloons and catheters and thrombolytic agents has been the standard procedure in declotting of thrombosed dialysis vascular accesses in many centers [1]. The thrombus from the clotted access can be dislodged during the procedure leading to embolization to the central veins and lungs. In fact, pulmonary embolism during thrombolysis of hemodialysis vascular accesses has been well documented [2–5]; however, occurrence of paradoxical embolism is very rare. On review of the literature up to July 2012, there have only been four case reports of paradoxical emboli secondary to thrombectomy of hemodialysis accesses [6–9]. 

## 2. Case Presentation

A 74-year-old black man with history of end-stage renal disease maintained on hemodialysis for five years, coronary artery disease, peripheral vascular disease, atrial fibrillation on anticoagulation with warfarin, pacemaker placement for symptomatic bradycardia 10 years ago, diabetes mellitus, hypertension, and colon cancer was referred to an outpatient vascular access center for thrombosed right forearm loop graft, which has been in use for three years. Thrombolysis was performed using 4 mg of alteplase and 8000 units of heparin, followed by mechanical thromboaspiration. An eighty percent stenosis at the genu of the graft was found which was dilated with a balloon catheter. No central venous stenosis was seen. 

Follow-up imaging of the entire access circuit after the thrombolysis demonstrated patency of the access. The patient's vital signs were stable throughout the procedure and afterwards, and his INR prior to thrombolysis was 2.0. After completion of the procedure, the patient was noted to be lethargic and aphasic with right-sided hemiparesis.

**Figure 1:**
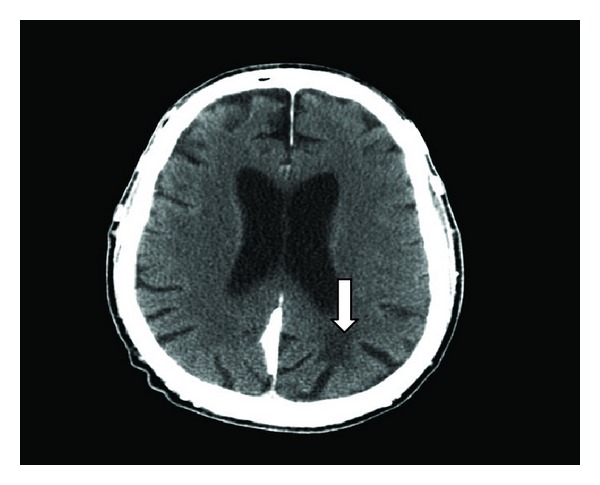
Nonenhanced CT scan shows a hypodensity in the left parietal lobe.

The patient was then transferred to the emergency room of our hospital. At time of admission, his blood pressure was 150/80 mmHg, heart rate was 60 beats/minute and regular. The neurological examination was significant for dysarthria, right ptosis, occasional vertical nystagmus, dense right upper motor neuron facial paresis, left tongue deviation, and right hemiplegia. His lungs were clear and he had a grade 2/6 systolic murmur at the left sternal border. The right forearm graft had a good bruit. His electrocardiogram showed ventricular pacing. Noncontrast head computed tomography (CT) revealed a hypodensity within the left posterior parietal lobe (Figure 1). A CT angiography of the brain and neck showed a small distal basilar filling defect near the left superior cerebellar artery and terminal bifurcation. The patient was not a candidate for intravenous tissue plasminogen activator due to therapeutic INR and he was past the 3-hour window.

A transthoracic echocardiogram with bubble study was performed due to strong suspicion of an embolic event, which showed right-to-left shunting, normal sized left atrium, and no evidence of intracardiac thrombus. It also revealed an elevated right ventricular systolic pressure and severe tricuspid regurgitation (an echocardiogram from 2010 showed similar findings of elevated right sided pressure and tricuspid regurgitation). A CT of the chest with pulmonary embolism (PE) protocol was not performed due to low clinical suspicion for pulmonary embolism because the patient had normal oxygen saturation on room air, no respiratory symptoms, and there were no electrocardiographic changes suggestive of pulmonary embolism. 

The patient was continued on anticoagulation with warfarin. He underwent physical rehabilitation and subsequently regained some functional ability in his right upper extremity. He was discharged to a subacute rehabilitation facility, but was readmitted one month later for sepsis and died during that admission. 

## 3. Discussion

The prevalence of end-stage renal disease patients on hemodialysis has been increasing for the past few years [10]. With this, there is also an expected increase in the number of complications from hemodialysis vascular accesses, which often include thrombosis and venous stenosis. Thrombosis can lead to failure of hemodialysis access in approximately 80% of cases [11, 12]. In one prospective study, up to 77% of arteriovenous grafts required a salvage procedure to maintain graft patency (thrombectomy, angioplasty, or surgical revision) after one year [13]. Percutaneous endovascular techniques are commonly used to declot the thrombosed accesses with high rate of success and low rate of complications [14]. 

The thrombus from a clotted dialysis access has a high likelihood of embolizing into the lungs, but symptomatic or clinically significant pulmonary embolism (PE) is rare [2–5]. Conditions such as pulmonary embolism, chronic lung disease, and Valsalva maneuver can increase the right heart and pulmonary artery pressures. When patent foramen ovale is present, an increase in right heart pressure can change the direction of the shunt (right to left) and result in paradoxical embolism. 

The incidence of patent foramen ovale (PFO) in the general population ranges from 25–30% [15]. Wu et al. have documented the safety of declotting procedures in 23 patients with known PFO who underwent 50 declotting procedures. There were no symptomatic paradoxical embolic events after the declotting procedure; however, this study is limited due to small sample size and retrospective nature [1]. Incidence of paradoxical embolism remains low due to low incidence of right-to-left shunts in patients with PFO, shunt only occurs intermittently during periods of volume overload or when intrathoracic pressure increases, and only a small volume of blood is shunted [6].

The timing of the stroke in relation to the procedure, an echocardiogram showing a normal sized left atrium with no intracardiac thrombus, bubble study revealing the presence of right-to-left shunting, and therapeutic INR on admission, are all highly suggestive that the stroke was a result of an embolus originating from the AVG. 

Paradoxical cerebral embolism can lead to catastrophic results. The thrombolytic technique which has the lowest risk for embolization remains to be investigated; maybe using a device with a basket can help catch dislodged clots to prevent thromboembolism. Clinicians and patients should be aware of paradoxical thromboembolism as a complication of vascular access procedures as morbidity and mortality rates are significantly increased [6–9].
